# Metabarcoding Analysis Reveals Microbial Diversity and Potential Soilborne Pathogens Associated with Almond Dieback and Decline

**DOI:** 10.3390/plants14152309

**Published:** 2025-07-26

**Authors:** André Albuquerque, Mariana Patanita, Joana Amaro Ribeiro, Maria Doroteia Campos, Filipa Santos, Tomás Monteiro, Margarida Basaloco, Maria do Rosário Félix

**Affiliations:** 1 MED—Mediterranean Institute for Agriculture, Environment and Development & CHANGE—Global Change and Sustainability Institute, IIFA—Instituto de Investigação e Formação Avançada, Universidade de Évora, Pólo da Mitra, Ap. 94, 7006-554 Évora, Portugal; mpatanita@uevora.pt (M.P.); joanaar@uevora.pt (J.A.R.); tomas.monteiro@uevora.pt (T.M.); margarida.fonseca@uevora.pt (M.B.); 2 MED—Mediterranean Institute for Agriculture, Environment and Development & CHANGE—Global Change and Sustainability Institute, Departamento de Fitotecnia, Escola de Ciências e Tecnologia, Universidade de Évora, Pólo da Mitra, Ap. 94, 7006-554 Évora, Portugal; mdcc@uevora.pt (M.D.C.); fs@uevora.pt (F.S.); mrff@uevora.pt (M.d.R.F.)

**Keywords:** *Prunus dulcis*, soilborne diseases, microbiome, *Neocosmospora rubicola* and the *Fusarium solani* complex, *Dactylonectria estremocensis*, *Plectosphaerella niemeijerarum*

## Abstract

Almond decline and dieback have become significant challenges in newly established orchards, with symptoms including internal necrosis, canker, and external gummosis. This work aims to explore the potential fungal and bacterial causative agents through metabarcoding and traditional culture plate isolation across six almond cultivars. Our results emphasize the multifactorial nature of almond decline and dieback, with possible co-infections by opportunistic fungi and bacteria playing a central role. Classical isolation identified 47 fungal species or genera, including *Diaporthe amygdali*, *Diplodia corticola*, *Phytophthora* sp., and several *Fusarium* species. Almond metabarcoding revealed a more diverse microbial community, highlighting the prevalence of soilborne pathogens such as *Neocosmospora rubicola*, *Dactylonectria estremocensis*, and *Plectosphaerella niemeijerarum*. Soil metabarcoding suggested that these pathogens likely originate from nursery substrates or soils shared with other crops, such as olives and vineyards, that serve as a source of inoculum. ‘Soleta’ generally presented lower richness when compared to the other tested cultivars, suggesting a higher degree of biotic stress and decreased plant resilience. This study highlights the value of integrating NGS approaches to comprehensively study complex diseases and the need for further research on pathogen interactions and cultivar susceptibility for the future development of new sustainable, targeted management strategies in almond orchards.

## 1. Introduction

The extensive production of almond (*Prunus dulcis*) is traditional in the Mediterranean landscape and presents significant economic, social, and cultural value in this region [1,2]. In 2022, Spain was the leader in total harvested area and the third largest almond producer worldwide, while Portugal registered a total of 63,880 ha, corresponding to the seventh position worldwide in this regard. The Portuguese production of shelled almonds grew over 500% in a twelve-year span, increasing from 7012 tons in 2010 to 46,215 tons in 2022 [3]. The expansion witnessed in Portugal is mainly led by investors in the Alentejo region, where olive and vineyard areas were progressively being replaced with irrigated intensive and super-intensive almond plantations. This was mainly due to the current attractive prices of this crop in international markets and the abundance of water from the Alqueva reservoir [4]. Furthermore, traditional, less productive almond cultivars have been abandoned and replaced by foreign cultivars more adapted to these intensive production systems [5]. The predominant cultivars produced in Alentejo include the French ‘Lauranne’ and the Spanish ‘Guara’, ‘Soleta’, ‘Belona’, and ‘Marinada’, among others. Besides producing more, these cultivars also have the added advantage of later flowering, allowing them to minimize production losses caused by the frequent late spring frosts in the region [6].

However, these newly installed almond orchards have been suffering significant production losses, which can be as high as 30% of total production. The overall health and vigor of the plants gradually deteriorate, leading to decline and dieback, and can end with the plant’s death. These complex conditions are known to be particularly associated with vascular/trunk diseases and stem cankers caused by several opportunistic pathogenic fungi [7,8,9,10].

Plant decline is generally used to describe their premature and progressive reduction of growth and vigor and can have multiple causal factors, including temperature, nutrient imbalance, soil structure, and pathogen invasion, among others. Dieback, on the other hand, is used to describe the progressive death of twigs and branches from the top down. Plant decline and dieback are closely associated, with dieback often serving as a sign of a broader ongoing decline process. While dieback is often represented by localized death, decline reflects the systemic response to the environmental stressor. Pathogen-induced dieback can develop when a disruption of the roots or vascular system occurs and is caused by opportunistic fungi, bacteria, viruses, or nematodes, responsible for several diseases that block or significantly reduce water and nutrient flow, leading to plant tissue death [11]. Excluding the diseases caused by nematodes and viruses, the group of diseases that can lead to dieback includes the following: vascular wilt—caused by fungi such as *Verticillium* spp. and *Fusarium* spp., and the bacteria *Ralstonia* spp., *Pseudomonas* spp., *Clavibacter* spp., *Erwinia* spp., and *Xylella* spp. [12,13]; trunk and stem cankers—caused by fungi such as *Botryosphaeria* spp., *Cytospora* spp., *Diaporthe* spp., *Diplodia* spp., *Dothiorella* spp., *Lasiodiplodia* spp., *Neofusicoccum* spp., and *Eutypa* spp., among others [14,15]; root and crown rots—mainly caused by soilborne oomycetes such as *Phytophthora* spp. and *Pythium* spp. and fungi like *Rhizoctonia* spp., *Sclerotinia* spp., *Armillaria* spp., *Cylindrocarpon* spp., and its related cylindrocarpon-like asexual states-nectria species [16,17,18]; bacterial blight—caused by *Pseudomonas* spp. and *Xanthomonas* spp. [19]; and wood discoloration and decay—caused by fungi such as *Phaeoacremonium* spp., *Phaeomoniella* spp., and *Fomitiporia* spp., among others [20].

Many of these microorganisms coexist in complex microbial communities within the host, often contributing to multiple overlapping diseases. These interactions can include antagonism, synergism, coexistence, mutualism, or cooperation [21]. For example, *Coniothyrium minitans* (also known as *Paraphaeosphaeria minitans*) is a highly specific mycoparasite that can act as a biocontrol of the soilborne plant pathogen *Sclerotinia sclerotiorum* while having little to no effect on other present microbial populations [22]. On the other hand, co-infection of *Botryosphaeria* and *Ilyonectria* species results in higher decline of young grafted field grapevines when compared to inoculation with only *Ilyonectria* sp. [23]. Similarly, it has previously been suggested that *Fusarium* spp. can aggravate grapevine trunk disease symptoms when inoculated with other known pathogens, such as *Dactylonectria macrodidyma* [24]. These examples illustrate the challenges in diagnosing and managing such diseases, where pathogens either work synergistically during co-infections or sequentially weaken plant defenses, resulting in accumulated stress and decline. Therefore, understanding host–multiple-pathogen interactions is crucial for the prediction of long-term dynamics of multiple disease outcomes [25].

Classical study of plant diseases focuses on the Petri dish isolation of fungi and bacteria, with selective or semi-selective media, where individual cultures are maintained based on specific growth characteristics. These can later be morphologically distinguished and/or specific PCR amplified and Sanger sequenced [26]. While foundational, this method has significant limitations, particularly in the study of complex diseases that involve multiple microbial origins. In vitro isolation often overlooks less dominant or slow-growing organisms, as fast-growing species can overshadow them in culture. Additionally, traditional isolation techniques can disrupt natural interactions between microbes, leading to an incomplete or biased view of the disease’s microbial ecology. Today, the study of complex polymicrobial diseases can benefit from culture-independent analyses such as those involving direct DNA sequencing within a sample, capturing the entire microbiome associated with the diseased tissue. DNA metabarcoding identifies species by sequencing all amplified sequences in a specific region (barcode) present in environmental samples, producing data on biodiversity, community composition, and ecological interactions. Next-generation sequencing (NGS) methods, such as DNA metabarcoding, have enabled high-throughput data collection of diverse microbial populations, revealing both dominant and rare organisms, including unculturable or morphologically identical species that are often too difficult to grow traditionally [21,27,28].

This work intends to explore the likely fungal and bacterial causative agents of the occurring events of almond dieback and decline through metabarcoding and traditional isolation in the newly established orchards of the Alentejo region.

## 2. Materials and Methods

### 2.1. Sampling Conditions

**Figure 1:**
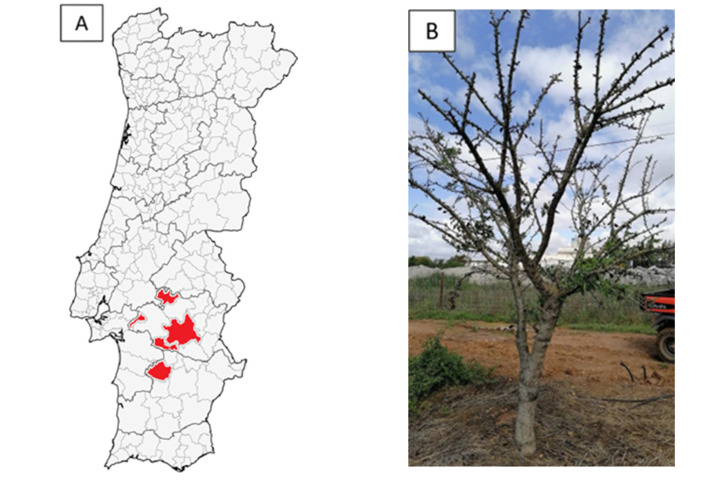

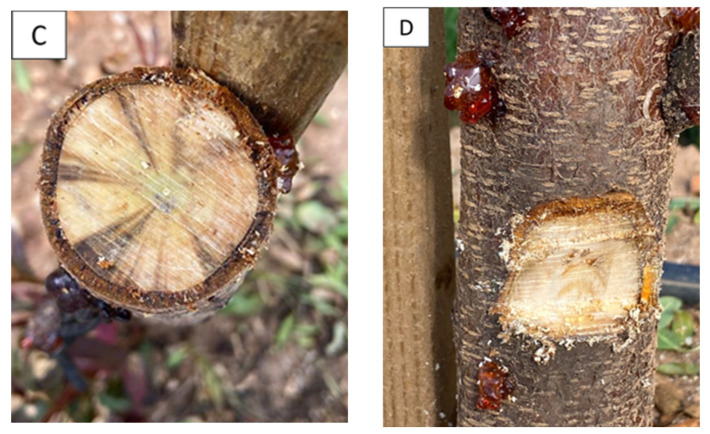
Visual symptoms of recent almond orchards in the Alentejo region. (**A**) Map highlighting in red the Alentejo municipalities with sampled orchards. (**B**) Almond tree showing extensive symptoms of decline and dieback. (**C**) Transversal cut of an almond tree showing symptoms of internal necrosis and wood decay. (**D**) Almond tree showing symptoms of canker disease leading to excessive gumming.

In the growing season of 2022, some newly installed (two- and/or three-year-old) almond orchards in the Alentejo region (Figure 1A) started to show overall symptoms of decline and dieback (Figure 1B), along with internal necrosis of the trunks (Figure 1C) and external gummosis (Figure 1D).

Trunk chunks, twigs, leaves, and roots were randomly sampled from a total of 44 trees presenting these symptoms from six of the most common almond cultivars in Portugal, ‘Soleta’ (seven samples), ‘Guara’ (seven samples), ‘Belona’ (seven samples), ‘Antoñeta’ (six samples), ‘Lauranne’ (13 samples), and ‘Makako’ (four samples). The samples were collected from 10 different orchards in the Alentejo region (Figure 1A); however, the exact locations are not revealed to conserve the anonymity of contributing orchards. A composite randomized soil sample was also obtained from each sampled orchard (*n* = 10) by collecting soil from several randomly selected locations within the orchard, which were then thoroughly mixed to create a single representative composite sample for analysis.

### 2.2. Sample Processing and Fungi Isolation

From the 44 collected samples, 12 were randomly selected, and small trunk and twig segments (each approximately 5 mm^2^) of these were placed on potato dextrose agar (PDA; VWR International, Leuven, Belgium, 39 g L^−1^) in 90-by-13 mm Petri dishes. The resulting plates were incubated at room temperature, and emerging colonies were transferred to newer PDA filled Petri dishes until a pure isolate was obtained.

### 2.3. DNA Extraction, PCR, and Sanger Sequencing of Plate Isolates

Isolates in the plates were transferred to mortars using a sterile scalpel blade and macerated in liquid nitrogen. Total DNA was extracted with the standard cetyl trimethylammonium bromide (CTAB) method. Quantity and quality were assessed with a Quawell Q9000 Series UV-Vis spectrophotometer (Quawell Technology, Sunnyvale, CA, USA). Amplification reactions were set up in a 50 µL final volume mixture consisting of 5 µL of DreamTaq Green Buffer (10×, Thermo Fisher Scientific, Waltham, MA, USA), 2 µL of each internal transcribed spacer (ITS) 1 (5′-TCCGTAGGTGAACCTGCGG-3′) and ITS4 (5′-TCCTCCGCTTATTGATATGC-3′) primer at 5 µM, 0.4 mM of a dNTP mixture (Thermo Fisher Scientific, Waltham, MA, USA), 2.5 U of DreamTaq DNA Polymerase (Thermo Fisher Scientific, Waltham, MA, USA), and 200 ng of the total DNA previously obtained. Amplification conditions encompassed an initial denaturation at 95 °C for 3 min, followed by 39 cycles of denaturation at 95 °C for 30 s, annealing at 55 °C for 45 s, and extension at 72 °C for 2 min, followed by a final extension at 72 °C for 10 min. Target PCRs were run in a LifeECO Thermal Cycler (Bioer, Hangzhou, China) along with no template controls. The presence of PCR products was confirmed by electrophoresis in a 1% agarose gel containing 1 µL of GreenSafe Premium (NZYTech, Lisboa, Portugal) and run at 90 V for 60 min. Amplified PCR products were purified using the NZYGelpure kit (NZYTech, Lisboa, Portugal) and sent for Sanger sequencing in both directions by STABVida (Caparica, Portugal). Sequences were manually trimmed in BioEdit (version 7.2.6), and the resulting sequences were blasted for homology using the nucleotide Basic Local Alignment Search Tool (BLAST) with default options at the National Center for Biotechnology Information website.

### 2.4. Sample DNA Extraction and Metabarcoding

Trunk portions of each of the 44 samples were ground together with the respective leaves and root pieces in an A10 basic IKA mill (IKA, Staufen, Germany), with the resulting powder macerated in liquid nitrogen with a mortar and pestle and stored at −20 °C. Total DNA was isolated from approximately 100 mg of the macerated plant tissue following the manufacturer’s instructions for the DNeasy Plant Pro kit (QIAGEN, Hilden, Germany) and stored at −20 °C until further use. DNA from soil samples was extracted using the DNeasy PowerSoil Pro kit (QIAGEN, Hilden, Germany). Quantity was assessed with a Quawell Q9000 Series UV-Vis spectrophotometer (Quawell Technology, Sunnyvale, CA, USA), and integrity was assessed using a 1.5% agarose gel electrophoresis. DNA of all samples was sent for sequencing at STABVida (Caparica, Portugal). The libraries were constructed using the primers ITS1f (5′-CTTGGTCATTTAGAGGAAGTAA-3′) and ITS2 (5′- GCTGCGTTCTTCATCGATGC-3′) for fungal detection and 16S_F (341F; 5′-CCTACGGGNGGCWGCAG-3′) and 16S_R (785R; 5′-GACTACHVGGGTATCTAATCC-3′) for bacterial detection. Library construction followed the Illumina 16S Metagenomic Sequencing Library preparation protocol (15044223 Rev. B). Quality control of the libraries was assessed using a Qubit 2.0 fluorometer (Thermo Fisher Scientific, Waltham, MA, USA) and the Qubit dsDNA BR kit. Paired-end sequencing was performed using the MiSeq reagent kit and conducted on a MiSeq PE300 platform, with the target read length set for 300 bp. Sequencing depth was up to 100,000 reads per sample, and an alpha rarefaction curve was performed for each sample to identify a reasonable amount of sequenced reads. Quality control of the sequenced data was performed using FastQC software (version 1.0.0).

### 2.5. Bioinformatic and Statistical Analyses

Post-sequencing analysis of the generated raw data was performed using QIIME2 (version 2024.2). Reads were trimmed for low-quality regions, dereplication events, and chimeras using the DADA2 plugin. The reads were organized in Features and classified by taxon using the scikit-learn classifier based on the SILVA database (release 138 QIIME) with a clustering threshold of 99% similarity for the 16S and based on the UNITE (release 9) database with a dynamic clustering threshold for the ITS. For classification, only OTUs with a minimum of 10 sequence reads were included. The final OTU tables were modified so that only the taxonomical levels of genus (level 6) and species (level 7) were considered for further analysis.

Resulting OTU tables were uploaded to the marker data profiling module of MicrobiomeAnalyst version 2.0 [29] along with the respective taxonomy tables and metadata. Standard data filtering for low counts and low variance was performed. Data normalization was also set to the standard options. Data were not rarefied nor transformed and were scaled using the total sum scaling method. Alpha-diversity profiling was estimated with Chao1 and Shannon diversity indexes, with a Welch T-test/ANOVA for multiple group comparisons, adjusted with the Benjamini-Hochberg procedure. Beta-diversity profiling was computed with PCoA as the ordination method, based on the Bray–Curtis dissimilarity at the feature level, and a PERMANOVA analysis was performed for the multiple pairwise comparisons, adjusted with the Benjamini–Hochberg procedure. The Core Microbiome prediction was executed at a feature level with a sample prevalence of 15% and relative abundance of 0.01%. Statistical significance was set at values lower than 0.05, and values between 0.05 and 0.1 were considered trends.

## 3. Results

### 3.1. Isolate Sanger Sequencing

A total of 47 fungal isolates were obtained from almond trees showing symptoms of decline and dieback, with over 50% belonging to the genera *Alternaria* and *Fusarium*. The complete list of fungal isolates is shown in Table 1.

### 3.2. Metabarcoding of the Fungal Communities in Almond Trees

For the ITS metabarcoding and prior filtering, a total of 2,891,651 read counts were obtained, and these were distributed among 762 OTUs (genus and species), with an average count per sample of 65,719. An Excel database was built (Supplementary Table S1) with information on the total list of OTUs, associated sample cultivar, and detailed read count.

Regarding known pathogenic fungi, the most abundant in terms of sequenced reads were *Neocosmospora rubicola* (present in 59.09% of samples), *Dactylonectria estremocensis* (68.18%), *Plectosphaerella niemeijerarum* (72.73%), *Phytophthora pisi* (20.45%), *Sclerotinia sclerotiorum* (45.45%), *Fusarium solani* (43.18%), *Cadophora luteo-olivacea* (15.91%), and *Truncatella angustata* (29.55%). The 50 OTUs represent approximately 5.73% of all sequenced reads, with *Neocosmospora rubicola*, *Dactylonectria estremocensis*, and *Plectosphaerella niemeijerarum* alone accounting for 3.46%.

**Figure 2:**
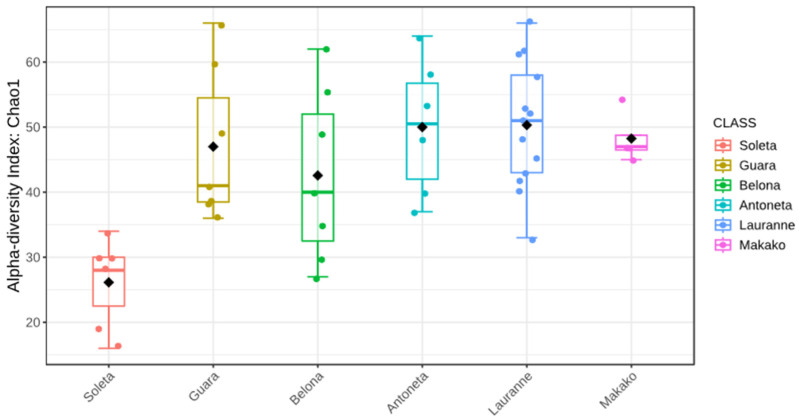
Boxplot illustration of the Chao1 alpha-diversity index from the ITS metabarcoding of six almond tree cultivars exhibiting decline and dieback. Black diamonds represent the group mean.

Alpha-diversity analysis using the Chao1 index set the ‘Soleta’ group apart from the other cultivars (Figure 2). This is confirmed in the multiple comparisons (Table 2) between this cultivar and ‘Guara’ (FDR = 0.009), ‘Belona’ (FDR = 0.049), ‘Antoñeta’ (FDR = 0.006), ‘Lauranne’ (FDR = 6.099 × 10^−5^), and ‘Makako’ (FDR = 4.888 × 10^−4^). However, using the Shannon index, no statistical difference was inferred between any two groups (*p* ≥ 0.05).

**Figure 3:**
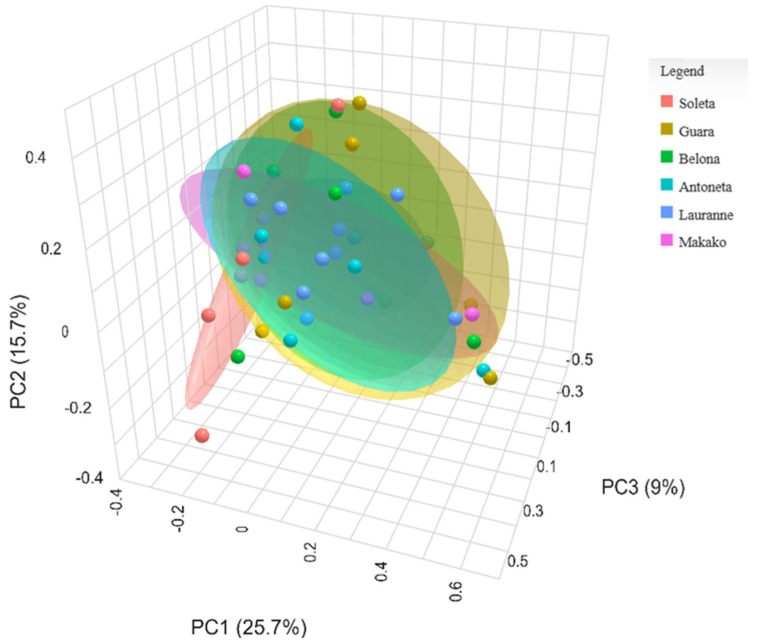
Principal Coordinates Analysis (PCoA) of the beta-diversity using the Bray–Curtis index from the ITS metabarcoding of six almond tree cultivars exhibiting decline and dieback.

Beta-diversity analysis revealed no clear clusters through the Principal Coordinates Analysis (PCoA) plot, with the first three PCs explaining 50.4% of the total variance (Figure 3). This is confirmed in the multiple comparisons test (Table 3), in which no statistical significance was obtained between any groups (*p* ≥ 0.05). The most distant groups were ‘Soleta’ and ‘Lauranne’, presenting an FDR of 0.060.

**Figure 4:**
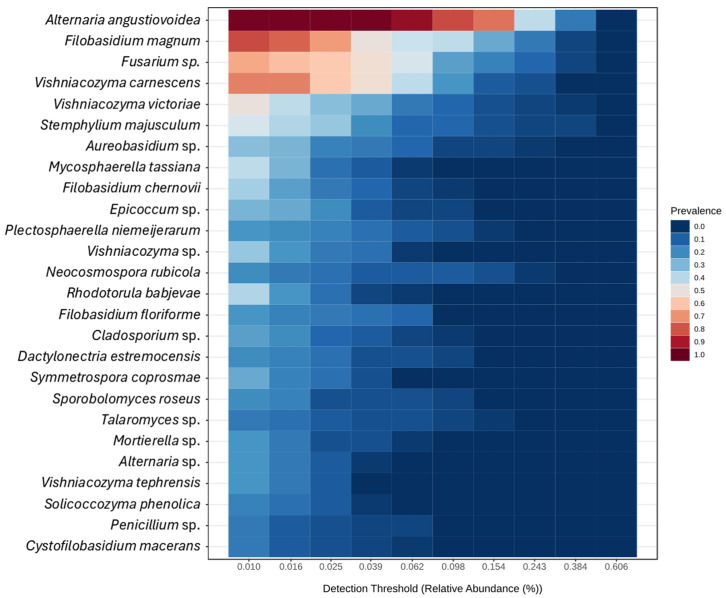
Heatmap of the core microbiome from the ITS metabarcoding of six almond tree cultivars exhibiting decline and dieback. Sample prevalence was set at 15%, and minimum relative abundance at 0.01%.

Regarding the overall core microbiome, a total of 26 OTUs were obtained, distributed between nine genera and 17 species (Figure 4).

### 3.3. Metabarcoding of the Bacterial Communities in Almond Trees

For the 16S ribosomal RNA metabarcoding and prior filtering, a total of 753,356 read counts were obtained, and these were distributed among 783 OTUs (genus and species), with an average count per sample of 17,937. An Excel database was built (Supplementary Table S2) with information on the total list of OTUs, associated sample cultivar, and detailed read count.

Regarding potential pathogenic bacteria, the most abundant in terms of sequenced reads were *Pseudomonas* sp. (present in 90.70% of samples), *Lonsdalea* sp. (4.65%), *Pantoea* sp. (76.74%), *Erwinia* sp. (9.30%), *Xanthomonas* sp. (20.93%), and *Curtobacterium* sp. (51.16%). A total of 11 OTUs were identified, representing approximately 23.69% of all sequenced reads, with *Pseudomonas* sp., *Lonsdalea* sp., and *Pantoea* sp. alone accounting for 18.83%.

**Figure 5:**
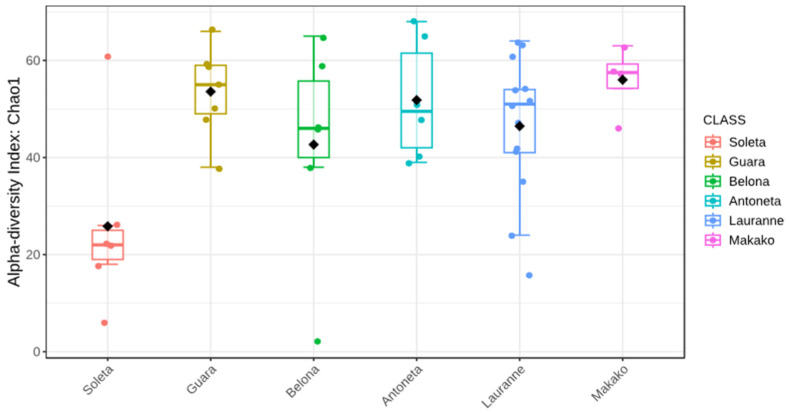
Boxplot illustration of the Chao1 alpha-diversity index from the 16S metabarcoding of six almond tree cultivars exhibiting decline and dieback. Black diamonds represent the group mean.

Alpha-diversity analysis using the Chao1 index showed the Soleta group with the lowest values among all tested cultivars (Figure 5). Although not statistically significant, the comparisons with ‘Guara’ (FDR = 0.093), ‘Antoñeta’ (FDR = 0.097), and ‘Makako’ (FDR = 0.093) showed a tendency toward the lower alpha diversity of ‘Soleta’ (Table 4). Using the Shannon index, no statistical difference was inferred between any two groups (*p* ≥ 0.05).

**Figure 6:**
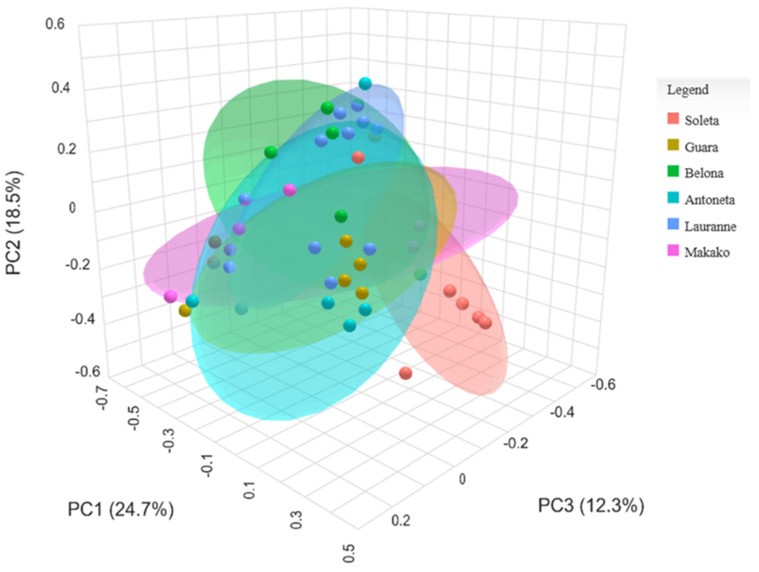
Principal Coordinates Analysis (PCoA) of the beta-diversity using the Bray–Curtis index from the 16S metabarcoding of six almond tree cultivars exhibiting decline and dieback.

Beta-diversity analysis revealed an isolated cluster of the ‘Soleta’ group through the Principal Coordinates Analysis (PCoA) plot, with the first three PCs explaining 55.5% of the total variance (Figure 6). This clustering was supported by the multiple comparisons test (Table 5), where the ‘Soleta’ group was statistically different from ‘Belona’ (FDR = 0.045) and ‘Lauranne’ (FDR = 0.015) and showed a trend toward difference with ‘Guara’ (FDR = 0.065) and ‘Antoñeta’ (FDR = 0.079). ‘Soleta’ vs. ‘Makako’ was the only ‘Soleta’ comparison that did not reach any statistical significance (FDR = 0.135). No statistical significance was obtained between any other pair comparisons (*p* ≥ 0.05).

**Figure 7:**
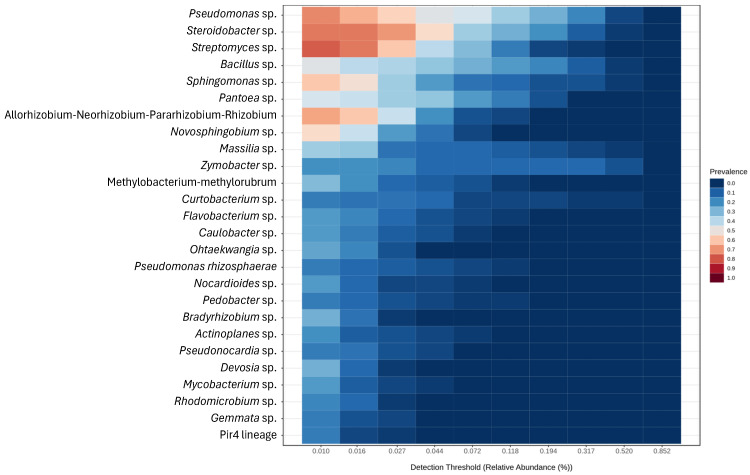
Heatmap of the core microbiome from the 16S metabarcoding of six almond tree cultivars exhibiting decline and dieback. Sample prevalence was set at 15%, and minimum relative abundance at 0.01%.

A total of 26 OTUs constitute the overall core microbiome, distributed between 25 genera and only one species (Figure 7).

### 3.4. Metabarcoding of the Soil Samples

For the ITS metabarcoding and prior filtering, a total of 879,944 read counts were obtained, and these were distributed among 686 OTUs (genus and species), with an average count per sample of 87,994, while the 16S yielded a total of 291,410 reads distributed among 826 OTUs, averaging 29,141 per sample. An Excel database was built (Supplementary Tables S3 and S4 for fungi and bacteria, respectively) with information on the total list of OTUs, associated sample names, and detailed read counts.

## 4. Discussion

To the best of our knowledge, this study is the first to investigate the microbial populations colonizing almond trees showing dieback and decline symptoms through NGS. Metagenomic studies on these plant diseases are scarce and usually performed in grapevines [30,31] and other stone fruit tree crops such as walnuts [32]. Current studies on these diseases are performed traditionally with plate isolation and have been able to identify associated pathogens such as *Botryosphaeriaceae* species, *Cytospora*, *Eutypa*, and several *Fusarium* species, among others [14,33,34,35,36,37].

In this study, we successfully characterized the microbial populations of recently installed almond trees, belonging to six different cultivars, that started displaying overall symptoms of dieback and decline, with associated internal necrosis, canker, and gummosis.

The ‘Soleta’ cultivar presented the lowest estimations of alpha-diversity regardless of the amplicon sequenced (ITS or 16S, Figure 1 and Figure 4). The Chao1 index reached a level of statistical significance (*p* < 0.05) between ‘Soleta’ and any other cultivar for the ITS, while the Shannon index did not achieve any statistical significance between any two groups. Similarly, the 16S sequencing revealed three pair comparisons with a tendency level of significance using the Chao1 index (‘Soleta’ vs. ‘Guara’, FDR = 0.093; ‘Soleta’ vs. ‘Antoñeta’, FDR = 0.097; ‘Soleta’ vs. ‘Makako’, FDR = 0.097), but none using the Shannon index. Since Chao1 specifically estimates richness based on the abundance of observed and rare taxa in the samples, while Shannon equates both richness and evenness, we can conclude that the distribution of existing species remains consistent across all samples despite the lower richness. The fact that ‘Soleta’ presented lower richness indicates that fewer unique taxa are present within this group, suggesting a higher degree of biotic stress and decreased plant resilience. Interestingly, beta diversity analysis revealed a more separate cluster of the ‘Soleta’, while the other cultivars were grouped more closely, suggesting a distinctive community structure of ‘Soleta’, particularly regarding the bacterial populations. These results may indicate a higher level of susceptibility of this cultivar to decline and trunk diseases, agreeing with personal observations by farmers who have reported a higher incidence of symptomatology in ‘Soleta’ trees compared to other cultivars. This hypothesis should be explored in future studies.

The possible emergence of new pathogenic species and the adaptation of old ones from other crops, such as vineyards and olive trees, are causing complex modern diseases in almond orchards that are not yet fully understood, and these are potentiated by climate change, global commerce, and other anthropogenic factors [38].

A total of 50 OTUs (genus and species of fungi) previously associated with trunk diseases and dieback in fruit trees were identified (Supplementary Table S1), with most of these primarily associated with root and crown rots, highlighting the central role of soilborne pathogens.

Studies on *Neocosmospora rubicola* are limited, but existing research highlights its pathogenicity in various crops. *Neocosmospora rubicola* is a recognized potato [39] and pitaya [40] pathogen causing stem rot that can sometimes develop into root rot. Species in this genus have previously been reclassified from the *Fusarium solani* group complex as members of *Neocosmospora* [41]. Given its ability to infect diverse hosts, similar to other *Fusarium* species, its presence in almond orchards warrants further investigation, particularly regarding its potential role in the development of root and crown rot symptoms.

*Dactylonectria estremocensis*, previously known as *Ilyonectria estremocensis*, is a common soilborne fungus responsible for causing black-foot disease in field nurseries and young vineyards. Nevertheless, it has been suggested a dual role of these fungi since they were also isolated from asymptomatic plants, indicating that they can be non-pathogenic endophytes in certain plants, acting as reservoirs of inoculum and causing future outbreaks in other crops/plants [42,43].

*Plectosphaerella niemeijerarum*, also known as *Plectosphaerella plurivora*, is a fungal pathogen causing necrosis of roots and crown in many plant hosts, including horticultural crops [44], basil, and parsley [45]. Although not extensively studied in almonds, grapevines, or olives, *Plectosphaerella niemeijerarum* might be contributing to the development of root and crown rots, and future studies should clarify this hypothesis.

*Phytophthora pisi* is a recognized soilborne pathogen for pea and faba bean, causing root and crown rot [46]. Research involving *Phytophthora pisi* in stone fruits is limited, with other species such as *Phytophthora cactorum*, *Phytophthora syringae*, *Phytophthora citricola*, *Phytophthora niederhauserii*, and *Phytophthora mediterranea* being more commonly reported [47,48,49].

*Sclerotinia sclerotiorum* is another soilborne plant pathogen known for infecting roots and causing stem and crown rot. It has evolved as an exceptionally versatile pathogen, capable of infecting a broad range of plant hosts from herbaceous to woody species due to its infection strategy, using general mechanisms such as necrotic growth, production of cell-wall degrading enzymes, and the secretion of oxalic acid to weaken defenses and further colonize plant tissues [50,51,52].

*Fusarium solani* is also a versatile pathogen capable of causing wilting and root, crown, and stem rots across a wide range of herbaceous and woody hosts [33]. In almonds, it has also been previously described to cause stem canker, gummosis, branch dieback, and wood discoloration [35].

In grapevines, the enrichment of *Fusarium* spp. in symptomatic trunk diseases vs. asymptomatic plants suggested that it may play an important role in grapevine trunk diseases (GTDs) development [24]. In our study, *Fusarium* spp. presented the third highest read count of all OTUs analyzed (only genus and species, Supplementary Table S1), possibly prompting the development of trunk diseases. We suggest that a similar pattern might be occurring with *Stemphylium majusculum* (present in 93.18% of samples), *Cristulariella depraedans* (6.81%), and *Mycosphaerella tassiana* (93.18%, also known as *Cladosporium herbarum*), which presented the 5th, 9th, and 18th highest abundance of reads, respectively, and resided within the core microbiome analysis performed (Figure 3). These species include known plant pathogens that affect multiple hosts, including herbaceous and woody species, and mostly cause foliar diseases such as leaf spots, blights, and necrotic lesions that lower overall photosynthetic activity and overall plant vigor, contributing to decline [53,54,55]. Furthermore, these species can act as stress-inducing agents, weakening the hosts and predisposing them to secondary infections by opportunistic trunk pathogens that lead to dieback and further decline.

Regarding the bacterial communities, only 11 OTUs (genus and species of bacteria) were found with potential pathogenic activity in stone fruit trees (Supplementary Table S2).

*Pseudomonas* species constitute a highly diverse group of bacteria with a wide host range, including plant pathogens, plant growth-promoting bacteria (PGPB), saprophytes, and also candidate biocontrol agents for canker and wilt diseases such as *Pseudomonas aeruginosa* AC17 [56] and *Pseudomonas fluorescens* WCS365 [57], respectively. Regarding the species with pathogenic potential to woody plants, *Pseudomonas syringae* should be highlighted, as it is described to cause bacterial canker and blight [58], and *Pseudomonas viridiflava*, which can cause spotting, blight, and stem rotting [59].

Evidence of *Lonsdalea* spp. associated with almond dieback and decline is limited, but these are closely associated with wood infections in various hosts. Some *Lonsdalea* species are emerging pathogens to oaks and other woody crops, particularly *Lonsdalea quercina*, which is responsible for blight, dieback in small twigs, branch cankers, and gummosis [60].

Similarly, several *Pantoea* species like *Pantoea agglomerans* and *Pantoea ananatis* have been described to cause multiple symptoms, including leaf spots, blight, trunk necrosis, and dieback in young pistachio and eucalyptus plantations [61,62].

*Erwinia* species are not widely recognized as primary pathogens in almond orchards, but some have been regarded as pathogenic in other hosts. *Erwinia rhapontici*, for example, has been reported to cause bacterial rot and shriveled stems in peach [63], while *Erwinia psidii* has been causing dieback and wilt in young eucalypt plantations [64].

The fact that no previous studies were performed on the phytosanitary situation of the soils where these almond plantations were installed suggests that these may have contributed to the transmission of pathogens from traditional plantations, such as vineyards and olive orchards, that find a common host in almond trees. A total of 24 OTUs (genus and species) of the aforementioned pathogenic fungi found in the almond trees were also detected in the soil, suggesting that the soils and/or nursery substrates might be acting as a source of inoculum. Examples of these are, by order of abundance, *Plectosphaerella niemeijerarum* (present in 90% of the soil samples), *Neocosmospora rubicola* (90%), *Fusarium solani* (100%), and *Dactylonectria estremocensis* (90%), which accounted for 5.18% of all sequenced reads. Present in all soil samples, *Fusarium* sp. was the OTU (genus or species) with the most sequenced reads overall, representing 7.63% and reinforcing the hypothesis of its association with the development of trunk diseases and overall decline.

Regarding the bacterial communities in the soil samples, a total of six genera of the potential pathogenic bacteria found in the almond trees were also detected in the soil. These are, by order of abundance, *Pseudomonas* sp. (present in 80% of the soil samples), *Pantoea* sp. (40%), *Serratia* sp. (10%), *Leifsonia* sp. (40%), *Xanthomonas* sp. (20%), and *Burkholderia* sp. (10%), which accounted for 3.90% of all sequenced reads.

The extensive microbial diversity and variability observed across all samples further challenge the traditional paradigm of ‘one pathogen, one disease’, highlighting the complexity of these interactions. Regarding dieback and decline and their associated symptoms, pinpointing the exact pathogen responsible is often unrealistic, since these are usually understood as a result of multiple stressors. Multiple pathogens can cause similar symptoms through distinct mechanisms, creating a spectrum of symptoms that are difficult to attribute to just one organism. Additionally, environmental stressors like drought or poor soil conditions can predispose plants to infection, allowing secondary pathogens to prosper, which further blurs the line between individual diseases. This is shifting how plant diseases are currently being studied from a singular pathogen approach toward understanding ‘disease complexes’, where multiple pathogens collectively contribute to plant decline [34]. The absence of a common pathogenic agent across all our almond samples highlights the diverse and multifactorial nature of the decline- and dieback-associated symptoms, emphasizing the complexity of this disease. Nevertheless, some more prevalent species should be highlighted, specifically *Neocosmospora rubicola* and the *Fusarium solani* complex, *Dactylonectria estremocensis*, and *Plectosphaerella niemeijerarum*. These potentially significant contributors to decline are hypothesized to engage in co-infections where their combined presence may amplify disease severity. The simultaneous presence of *Dactylonectria estremocensis*, *Plectosphaerella niemeijerarum*, and either *Neocosmospora rubicola* or *Fusarium solani* was detected in 47.73% of almond samples, and 88.64% had the presence of at least one of these species. Future research should prioritize detailed investigations into the pathogenic potential of these organisms independently and collectively. The bacterial results obtained from metabarcoding highlighted a distinct limitation in taxonomic resolution, with most identifications constrained to the genus level. This imposes additional challenges in drawing conclusions regarding the role of bacteria in the almond decline and dieback complex. Among the genera detected, *Pseudomonas* and *Pantoea* were some of the most abundant and prevalent. Both genera are known to include species capable of opportunistic pathogenicity or acting as endophytes, which can shift to a pathogenic role under favorable environmental conditions or host stress. However, given the lack of species-level resolution, their precise contributions remain speculative and require further complementary studies.

The classical isolation approach also revealed significant fungal diversity, with 47 distinct fungal species or genera successfully cultured from symptomatic almond samples. Among these are known pathogens such as *Diaporthe amygdali* and *Diaporthe foeniculina*, responsible for causing twig canker in a *Prunus* species [65] and participating in the grapevine trunk disease complex [66], respectively. Other isolated pathogens include four *Fusarium* species (*F. brachygibbosum*, *F. equiseti*, *F. oxysporum*, and *F. solani*), *Phytophthora* sp., and *Diplodia corticola*. *Diplodia corticola* has also previously been associated with almond decline [34]. These, with the exception of the *Fusarium solani* and *Phytophthora* sp., were found to be underrepresented in the metabarcoding analysis. Nonetheless, the metabarcoding analysis proved to be a more comprehensive approach, uncovering a broader diversity of fungal taxa associated with almond decline and dieback than the classical isolation method. This highlights its importance in studying disease complexes where multiple pathogens are involved, as it allows for the detection of hard-to-culture and low-abundance species that may be overlooked using traditional techniques. However, the classical method has the advantage of occasionally providing deeper taxonomic resolution, allowing researchers to distinguish species-level identities that may not be achievable with metabarcoding alone. Furthermore, this approach is essential for targeted future studies such as functional applications, pathogenicity testing, biocontrol screening, and the development of new diagnostic tools [67]. Together, these complementary approaches highlight the value of integrating advanced high-throughput molecular techniques with classical isolation to achieve a holistic understanding of microbial communities.

## 5. Conclusions

This study presents a comprehensive analysis of the microbial communities associated with almond dieback and decline in newly established orchards in the Alentejo region of Portugal. Through the integration of classical isolation methods and 16S and ITS metabarcoding, we identified a wide diversity of taxa potentially linked to these symptoms. Our results indicate that almond decline and dieback are not caused by a single pathogen but represent a complex disease driven by multiple stressors, including co-infections by opportunistic fungi and possibly bacteria. Classical isolation revealed 47 fungal species or genera, including known pathogens such as *Diaporthe amygdali*, *Diplodia corticola*, *Phytophthora* sp., and multiple *Fusarium* species. On the other hand, metabarcoding provided a broader overview, with a total of 50 OTUs (genus and species) identified among pathogenic fungi previously associated with trunk diseases and/or dieback. *Neocosmospora rubicola* and the *Fusarium solani* complex, as well as *Dactylonectria estremocensis* and *Plectosphaerella niemeijerarum*, were highly prevalent across the symptomatic almond samples but were found underrepresented or absent in the classical approach. This highlights the complementary nature of these methodologies, with metabarcoding offering insights into the full microbial spectrum, including hard-to-culture and low-abundance organisms. The frequent detection of these soilborne pathogens, along with their detected presence in soil samples, suggests that nursery substrates or contaminated soils may act as sources of inoculum. This study also emphasizes the importance of understanding the potential pathogenic roles of abundant bacterial genera such as *Pseudomonas* and *Pantoea*, which require further investigation due to the limited taxonomic resolution.

Cultivar-specific analyses revealed differences in the microbial communities, with ‘Soleta’ generally presenting lower richness when compared to the other tested cultivars. This indicates that fewer unique taxa are present within this group, suggesting a higher degree of stress and decreased plant resilience. Such results highlight the importance of cultivar-specific studies to identify cultivars with heightened vulnerabilities or potential resistance traits. These data are essential for guiding planting recommendations and disease management strategies. Future development of effective management strategies will need to address soil health and pathogen suppression to mitigate the impact of these complex diseases on almond orchards.

## Figures and Tables

**Table 1:** Taxonomy and isolation frequency of internal transcribed spacer (ITS) characterized fungi with greater than 99% homology match from almond trees exhibiting decline and dieback.

Family	Genus/Species
Dothioraceae (2)	*Aureobasidium pullulans* (2)
Diaporthaceae (5)	*Diaporthe amygdali* (4)
	*Diaporthe foeniculina* (1)
Trichocomaceae (6)	*Aspergillus* sp. (1)
	*Penicillium* sp. (1)
	*Penicillium citrinum* (4)
Pleosporaceae (15)	*Alternaria* sp. (10)
	*Alternaria alternata* (4)
	*Alternaria tenuissima* (1)
Nectriaceae (13)	*Fusarium brachygibbosum* (5)
	*Fusarium equiseti* (2)
	*Fusarium oxysporum* (3)
	*Fusarium solani* (3)
Hypocreaceae (1)	*Trichoderma virens* (1)
Didymellaceae (1)	*Stagonosporopsis* sp. (1)
Pythiaceae (1)	*Phytophthora* sp. (1)
Apiosporaceae (1)	*Arthrinium* (1)
Botryosphaeriaceae (1)	*Diplodia corticola* (1)
Cystobasidiaceae (1)	*Cystobasidium* sp. (1)

Taxonomic rankings from family to species are indicated, followed by isolation frequency in parentheses. Isolation frequency is the count of samples; each taxon was isolated from a possible 12 samples.

**Table 2:** Multiple pairwise comparisons of alpha-diversity indexes Chao1 and Shannon from the ITS metabarcoding between six almond tree cultivars exhibiting decline and dieback.

Comparison Pair	Statistic		*p*-Value		FDR	
	Chao1	Shannon	Chao1	Shannon	Chao1	Shannon
Soleta vs. Guara	−4.104	−0.961	0.002	0.361	0.009	0.563
Soleta vs. Belona	−2.968	−1.497	0.016	0.165	0.049	0.496
Soleta vs. Antoñeta	−4.871	−2.967	0.001	0.017	0.006	0.138
Soleta vs. Lauranne	−6.661	−2.628	4.065 × 10^−6^	0.018	6.099 × 10^−5^	0.138
Soleta vs. Makako	−7.054	−2.377	6.518 × 10^−5^	0.068	4.888 × 10^−4^	0.339
Guara vs. Belona	0.663	−0.355	0.52	0.729	0.747	0.781
Guara vs. Antoñeta	−0.487	−1.507	0.636	0.16	0.795	0.496
Guara vs. Lauranne	−0.635	−0.83	0.539	0.427	0.747	0.582
Guara vs. Makako	−0.256	−1.226	0.804	0.257	0.862	0.552
Belona vs. Antoñeta	−1.136	−1.221	0.28	0.248	0.601	0.552
Belona vs. Lauranne	−1.368	−0.447	0.202	0.664	0.505	0.766
Belona vs. Makako	−1.062	−0.948	0.321	0.376	0.601	0.563
Antoñeta vs. Lauranne	−0.061	1.028	0.952	0.331	0.953	0.563
Antoñeta vs. Makako	0.373	0.144	0.72	0.89	0.831	0.89
Lauranne vs. Makako	0.617	−0.713	0.548	0.51	0.747	0.638

**Table 3:** Multiple pairwise comparisons of the beta-diversity index from the ITS metabarcoding between six almond tree cultivars exhibiting decline and dieback.

Comparison Pair	F-Value	R-Squared	*p*-Value	FDR
Soleta vs. Guara	2.298	0.161	0.028	0.19
Soleta vs. Belona	1.856	0.134	0.073	0.274
Soleta vs. Antoñeta	1.789	0.14	0.038	0.19
Soleta vs. Lauranne	2.611	0.127	0.004	0.06
Soleta vs. Makako	1.663	0.156	0.111	0.333
Guara vs. Belona	0.514	0.041	0.887	0.943
Guara vs. Antoñeta	0.502	0.044	0.869	0.943
Guara vs. Lauranne	0.733	0.039	0.701	0.943
Guara vs. Makako	0.319	0.034	0.936	0.943
Belona vs. Antoñeta	0.611	0.053	0.844	0.943
Belona vs. Lauranne	0.55	0.03	0.943	0.943
Belona vs. Makako	0.443	0.047	0.92	0.943
Antoñeta vs. Lauranne	0.812	0.046	0.675	0.943
Antoñeta vs. Makako	0.457	0.054	0.924	0.943
Lauranne vs. Makako	0.763	0.048	0.737	0.943

**Table 4:** Multiple pairwise comparisons of alpha-diversity indexes Chao1 and Shannon from the 16S metabarcoding between six almond tree cultivars exhibiting decline and dieback.

Comparison Pair	Statistic		*p*-Value		FDR	
	Chao1	Shannon	Chao1	Shannon	Chao1	Shannon
Soleta vs. Guara	−3.332	−2.232	0.012	0.048	0.093	0.226
Soleta vs. Belona	−1.425	−0.8	0.185	0.446	0.407	0.652
Soleta vs. Antoñeta	−2.862	−2.138	0.019	0.06	0.097	0.226
Soleta vs. Lauranne	−2.401	−2.372	0.043	0.038	0.162	0.226
Soleta vs. Makako	−3.601	−2.684	0.009	0.033	0.093	0.226
Guara vs. Belona	1.124	0.868	0.301	0.409	0.502	0.652
Guara vs. Antoñeta	0.285	0.349	0.782	0.734	0.782	0.847
Guara vs. Lauranne	1.334	0.152	0.2	0.882	0.407	0.882
Guara vs. Makako	−0.488	−0.729	0.639	0.491	0.737	0.652
Belona vs. Antoñeta	−0.885	−0.674	0.403	0.521	0.575	0.652
Belona vs. Lauranne	−0.382	−0.821	0.714	0.438	0.764	0.652
Belona vs. Makako	−1.368	−1.375	0.217	0.207	0.407	0.62
Antoñeta vs. Lauranne	0.832	−0.235	0.421	0.818	0.575	0.876
Antoñeta vs. Makako	−0.676	−1.089	0.518	0.323	0.648	0.652
Lauranne vs. Makako	−1.762	−0.93	0.106	0.393	0.317	0.652

**Table 5:** Multiple pairwise comparisons of the beta-diversity index from the 16S metabarcoding between six almond tree cultivars exhibiting decline and dieback.

Comparison Pair	F-Value	R-Squared	*p*-Value	FDR
Soleta vs. Guara	2.61	0.192	0.013	0.065
Soleta vs. Belona	3.324	0.249	0.006	0.045
Soleta vs. Antoñeta	2.596	0.206	0.021	0.079
Soleta vs. Lauranne	4.628	0.214	0.001	0.015
Soleta vs. Makako	2.075	0.206	0.045	0.135
Guara vs. Belona	1.143	0.094	0.29 8	0.528
Guara vs. Antoñeta	0.315	0.028	0.918	0.918
Guara vs. Lauranne	1.15	0.06	0.315	0.528
Guara vs. Makako	0.695	0.071	0.579	0.62
Belona vs. Antoñeta	1.235	0.11	0.237	0.528
Belona vs. Lauranne	0.935	0.052	0.519	0.62
Belona vs. Makako	0.89	0.1	0.519	0.62
Antoñeta vs. Lauranne	1.114	0.061	0.317	0.528
Antoñeta vs. Makako	0.798	0.091	0.555	0.62
Lauranne vs. Makako	0.969	0.061	0.475	0.62

## Data Availability

The produced and analyzed datasets in this study are included within the main article and/or its Supplementary Files. All other data are available from the corresponding author upon reasonable request.

## Supplementary Materials

The following supporting information can be downloaded at: https://www.mdpi.com/article/10.3390/plants14152309/s1. Supplementary Table S1: Database of the ITS metabarcoding of the almond samples; Supplementary Table S2: Database of the 16S metabarcoding of the almond samples; Supplementary Table S3: Database of the ITS metabarcoding of the soil samples; Supplementary Table S4: Database of the 16S metabarcoding of the soil samples.
